# Validating a willingness to share measure of a vignette experiment using real-world behavioral data

**DOI:** 10.1038/s41598-025-92349-2

**Published:** 2025-03-18

**Authors:** Zoltán Kmetty, Ádám Stefkovics

**Affiliations:** 1 HUN-REN Centre for Social Sciences, CSS-RECENS Research Group, Budapest, 1097 Hungary; 2 Department of Sociology, Faculty of Social Sciences, Eötvös Loránd University, Budapest, Hungary; 3 Harvard University, IQSS, Cambridge, MA USA

**Keywords:** Vignette experiment, Survey, Validation, Data donation, Human behaviour, Computational science

## Abstract

Willingness measures are widely employed in vignette and conjoint experiments. However, the predictive validity of these measures relies on the degree to which expressed intentions correspond with behavior in the real world. This study uses a unique research design that enables to validate an intention measure. In a 2022 online survey, respondents expressed their willingness to donate their social media data in hypothetical scenarios. Nine months later the same respondents were invited to participate in a genuine data donation study. The results show that the correlation between willingness and actual donation was moderate ($\eta$: 0.28). Additionally, the determinants of self-reports and sharing behavior did not overlap. The willingness to donate was more strongly associated with softer attitudinal variables, whereas actual data sharing was linked more closely to harder demographic variables. These findings challenge the predictive validity of willingness to share measures and highlight the need for realistic scenarios in survey experiments, minimizing participants’ perceived risks of the task in question, and addressing social desirability bias.

## Introduction

Vignette and conjoint experiments are extensively utilized in the field of social sciences^1^. These methodologies aim to extract individuals’ expressed preferences in hypothetical situations and investigate their decision-making processes when faced with complex choices involving multiple dimensions. The attributes’ values are purposely altered randomly across participants and tasks. This intentional modification allows the researcher to determine the significance of each attribute in affecting the final choice or assessment. Advocates of such experiments contend that these experimental setups can reduce or even eliminate the discrepancy between surveys and reality, as they simulate actual decision-making tasks.

However, the predictive validity of these outcome measures in survey experiments are only valid externally to the extent that stated intentions align with real-world behavior^2,3^. Some theoretical considerations indeed suggest the opposite. First, the theory of planned behavior^4^ suggests that a low level of behavioral control or high personal risk makes intention-behavior relation weaker. Consequently, intentions may map real-world behavior more closely in low-risk situations^5^. Second, intentions measured in surveys are prone to “hypothetical bias”^6,7^. Situations depicted in vignettes inherently simplify and abstract real-world conditions, diminishing behavioral realism. Third, self-reported measures of intentions are susceptible to multiple cognitive biases, such as socially desirable responding^8^, satisficing^9^, or acquiescence^10^. Such respondent tendencies can lead to divergences from “true” actions. Nonetheless, even if reported intentions weakly predict behavior, examining what drives behavioral intentions may still help in understanding what determines human action (which is often the goal of survey experiments^1^).

The current state of research on the predictive validity of the outcomes of vignette and conjoint experiments is mixed and inconclusive. As demonstrated by Treischl & Wolbring^1^, one group of studies shows that estimates of vignette and conjoint experiments align well with behavioral data^11,12^, another group of studies reported low predictive validity^13–15^, and lastly, a third group of studies yielded mixed findings^2,5^. The latter studies suggest that while self-reported intentions and actual behaviors may differ in frequency, survey experiments could potentially infer behavioral determinants from expressed intentions. The inconsistencies in the literature are likely attributable to the use of varying measures across studies. The measures include willingness to accept lower wages^11^, willingness to hire black and white ex-offenders^15^, support for immigrants receiving citizenship^12^, aggressive responses in driving situations^13^ and responses to business requests for incorporation^14^.

In light of the inconsistencies yielded by prior research, further research is needed. Aside from mixed evidence, previous approaches exhibited certain methodological limitations. Some of them used field^5,13,14^ and natural experiments^12^ to validate survey experiments. These methods are often criticized due to the incomparability between the samples used in the factorial survey and the behavioral benchmark^5^. Other studies^11^ used self-reported behavioral measures as reference points, which might suffer from biases. To overcome these limitations this study uses a within-person comparison with real-world decisions (see^15,16^ for similar approaches).

We use a unique dataset that allows us to compare the outcomes of a vignette experiment (which assessed willingness to participate in a social media data donation study) to actual participation in a real data donation study involving the same survey respondents. The case of data donation offers a growing and important area to study. Since accessing digital social media data has recently become difficult, researchers are increasingly turning their attention to data donation approaches^17,18^, particularly data donation via data download packages (DDPs). DDPs comprise historical user data from social media, including behavioral, textual, media, and location facets. Users can access their DDPs, and due to their research value, scientists are gravitating towards it. In data donation, participants are procured using survey techniques and asked to share their DDPs with researchers, ensuring informed consent. Notably, sharing DDPs is considered a burdensome and sensitive task^19,20^ which may influence the validity of willingness to share measures^21^. Several earlier studies used vignette experiments to understand people’s willingness to share social media data (e.g.,^16,20,22,23^, but only one contrasted their findings with behavioral data. Keutsch et al.^16^ asked survey respondents at the end of the survey if they were open to donating some of their Facebook data. Although approximately 80% of the participants expressed their willingness to donate their data, only about one-third actually managed to complete the donation process successfully. The determinants of willingness and actual donation, however, were fairly consistent.

In this study, we compare responses from participants in an online vignette experiment conducted in Hungary in 2022, which explored the factors influencing willingness to donate social media data, against their actual donation behavior. We ask two research questions: What is the correlation between willingness and actual participation? Are the determinants of willingness and actual participation the same or are there differences?

## Materials and methods

### Data for the vignette experiment

Our research is built upon a 2022 vignette experiment survey^24^. This research aimed to understand how best to design a data donation study. The survey was administered by an online polling company, NRC, on a non-probability access panel. The NRC panel consists of more than 100,000 people. Compared to the general population of Hungary, individuals with a high level of education and from bigger cities are overrepresented in the panel. We used a quota sampling method (with quotas for gender, age, and geographical region) to ensure equal representation. The company gives regular incentives for the respondents of their studies. Altogether 1,000 respondents participated in the study. The fieldwork was carried out between 11-25, May 2022.

In this study, we applied a mixed factorial vignette design. Respondents had to evaluate multiple situations (called vignettes) in which we manipulated various dimensions of fictional research. At each vignette, they had to provide the likelihood of their willingness to donate their digital data in such research on a 0 to 10 scale. These manipulated dimensions of the fictional research are shown in Table 1 (an example of the vignettes is available in the SI).

**Table 1:** Manipulated dimensions and their levels in the survey experiment.

Dimension	Levels
Platform	Facebook
	Facebook and Google
	Facebook and other social media sites you use
	Facebook, Google, and other social media sites you use
Range of data	All, except: private messages
	All, except: private messages and location
	All, except: private messages and photo, videos
	All, except: private messages, and photo, videos, and location
Time to download/upload data	Less than an hour
	More than an hour
Incentives	3000 HUF (8.5 USD)
	5000 HUF (14.3 USD)
	10,000 HUF (28.6 USD)
Additional report	Yes
	No

Altogether we had 192 possible combinations of the dimensions in the following way: 4 [platform] * 4 [range of data] * 2 [time] * 3 [incentive] * 2 [report]. In the study, we created 16 decks (packages of vignettes) and assigned 12 vignettes to each deck (16 * 12 = 192). Thus, one respondent had to evaluate 12 different situations. With the expected (and then realized) 1000 respondents we had around 67 respondents per deck.

### Real-world behavioral data

Building on the results of the vignette experiment, we launched a broadly focused data donation study in 2023. The main objective of the research was to collect social media and digital trace data from a representative sample of internet users across multiple online platforms. The data collection in the project started in February 2023 and ended on 18th June 2023. From the respondents’ view, this was a three-phase procedure that consisted of

1. a short questionnaire with eligibility questions and a consent form,
2. downloading and uploading their data to the project’s website and
3. filling out a detailed survey questionnaire.

For each respondent, the process started with an invitation email from the data collection company (NRC). This email contained information about the project’s aims and incentives and a direct link to the project’s dedicated webpage. The materials are available in the Supplementary Information (Figs. S1–S3).

First, respondents filled out a short screening questionnaire (1). This allowed us to filter out respondents who do not have Google and Facebook profiles or do not use them regularly. These two digital data sources were mandatory for data donation. In this step, the respondents had to read a detailed research description and accept the consent form. Without expressing consent, the respondents could not move forward.

If the respondent was eligible to continue (2), they were presented with a webpage containing detailed guides for each platform (Google, Facebook, Instagram, Twitter, and TikTok). These guides contained step-by-step instructions on exporting and downloading information from these platforms, along with a video tutorial with the same content. Once the respondent downloaded their data onto their computer, they had to upload the file(s) for each platform without opening or extracting them to the project’s website. Uploading data for Google and Facebook was mandatory. However, those who uploaded their Instagram, TikTok, and Twitter profiles would receive more incentives.

After completing the data upload, participants were asked to complete a 30-minute questionnaire covering different topics (3). The questionnaire included questions on social status, leisure activities, news consumption, and political attitudes. At the end of the data donation research, 758 complete queries were completed.

In the first step of the data donation research outlined above, a call for research was sent to those who had participated in the 2022 vignette experiment study. Based on the identifiers provided by the research company, the respondents in the two surveys could be linked. For those respondents who completed both the 2022 questionnaire and the 2023 study, it was possible to examine whether the same people participated along the main demographic variables (gender, age). Three respondents had a difference in demographics, which was less than two percent of the linked people. These three people were removed from the analysis. We cannot do this check for the complete dataset, but even there, we cannot assume a discrepancy of more than two percent, which should not pose a severe problem for the validity of our data. For respondents participated in both studies, we can thus see what they answered to the hypothetical participation question and whether they ended up participating in a very similar research to the one we outlined for them in the vignette study. In this study, we focus only on those from the 2023 data donation study who also participated in the 2022 survey. We use the data from the 2022 survey in our analysis and only include one item from the 2023 study: how far the respondent has got in the study process.

In our current analysis, we excluded respondents who are not regular Facebook users and/or do not have a Google account because they were excluded from the data donation study. We also excluded respondents who were not members of NRC’s online panel at the start of the second study. We used a binomial logistic model to analyze whether there were any systematic differences between those who left the online panel and those who stayed members (see Fig. S4 in the SI). We only found weak effects; the R^2^ Tjur was lower than three percent. We found three significant variables: males, people with lower education, and younger people left the panel with a higher probability. However, we did not find any difference regarding the willingness to participate in the hypothetical data donation study. The final sample size was 769.

The data donation study was conducted with the IRB approval of Centre for Social Science Ethical Board (1-FOIG/130-37/2022) and with the informed consent of the participants. The Centre for Social Science IRB waived the ethical review of the preliminary study, as only anonymized data was processed. The data collection company collected consent forms for all the research participants. The study adhered to the ethical tenets of the Declaration of Helsinki.

### Analytical strategy

In the vignette experiment, twelve hypothetical situations were presented to the participants, and they were asked about their willingness to participate in such type of data donation research. In the original study^24^, we tested in many ways to see if there was any fatigue effect because of the high number of vignettes. However, we did not find any differences in the answers of the first and second half of the vignettes. Due to later practical considerations, none of the experiment scenarios perfectly matched the design we eventually followed for the 2023 study, but several variations were very close to the final implementation. For this study we measured which hypothetical vignettes were close to the actual data donation prompt. In the vignette study, two factors influenced willingness to participate: whether the donation was requested for Facebook data only or other platforms and the monetary incentive offered to the participants. The results of the vignette experiment showed that the latter effect was more robust and the former effect weaker^24^. We considered those the closest vignettes, where the offered incentive was 10,000 HUF (around 25 euros). We can not match the platform selection with the vignettes, as the implemented version (Facebook + Google mandatory, other platforms optional) was not included in the vignettes. We calculated the mean willingness score of these closest vignettes. We considered this the respondent’s estimated willingness score for the actual query. To test the robustness of the results, we also calculated a mean willingness score with all vignettes. These robustness calculations will be discussed in the results section. In the first step of the analysis, we compare the answer to the willingness question and whether the respondent (1) consented to participate in the second study and (2) successfully donated their data. We then use ANOVA analysis to show the willingness value of the respondents who dropped out at different stages. These first two analyses will answer the first research question about how willingness and participation are related at the respondent level. Our second research question focuses on whether the determinants of willingness and actual participation differ. To answer this, we run three logistic regression models. We used logistic models in the study as some dependent variables were close to the 20-80 ratio, where logistic models perform better than linear models^38^. However, for a robustness check, we also ran our analysis with linear probability models. These models are available in the supplementary. In the first model, the dependent variable is a dummy variable formed from the willingness to participate measure of the vignette experiment. We contrast this model with two different levels of participation in the second study. The first level consists of those who consented to participate in the data donation study. This means they got the invitation, went to the research platform, read the consent form, and accepted it. So, they show a willingness to participate. However, not all of them donated their data. So, in the second level, we focus on those who successfully donated their data. They uploaded valid digital data and completed a 30-40-minute questionnaire. We used several independent variables in the analysis. The first set of questions were related socio-demographic information. Following the original study^24^ we included gender, highest level of education, age (age variable was divided by 10 for ease of interpretation) and subjective wealth. Age is a key determinant of both digital technology use and attitudes toward digital technologies^25^. It is also likely to correlate with willingness to participate in digital data collection, as younger generations typically exhibit higher participation willingness^20,26^, which tends to decline after the age of 50^27^. Other socioeconomic factors, such as education, settlement type, and region, were also associated with willingness to participate^25,28^. Willingness to share data may also depend on the frequency of platform or device usage. Heavy users are generally assumed to be more open to data sharing^29^. For example, Silber et al.^20^ found that greater platform usage increased the likelihood of sharing social media data, although this effect did not extend to health app data. Thus we included the frequency of social media usage and the number of social media platforms used by the respondent. Another factor is privacy concerns. Previous studies consistently demonstrate that privacy and security concerns reduce willingness to participate in digital data sharing (e.g.,^30,31^). These concerns are frequently cited as the primary reasons for reluctance to engage in digital data sharing. To address this we used the Internet Users’ Information Privacy Concerns (IUIPC) scale to measure privacy concerns. Participating in a data donation study is an active task that requires a baseline level of digital literacy^32^, potentially placing a greater burden on individuals with lower technical skills. To control for this, we measured the Affinity for Technology Interaction Scale. Lastly, Psychological traits may also influence willingness to participate in data donation studies. Some studies suggest that conscientiousness can increase participation, while introversion is associated with a greater likelihood of sharing GPS data^26^, although other studies^20^ found no significant positive effects of these traits on sharing social media or health data. To measures psychological traits we used the Big Five (BFI-S) inventory. See a more detailed discussion of theoretical assumptions behind the inclusion of these covariates in^24^. A detailed description of the independent variables is available in the SI.

There were no missing values in the willingness to participate measure, only in the independent ones. In 28% of the cases, there was at least one missing variable. We applied multiple imputations to handle these missing values. We included all the independent variables in the imputation process and used predictive mean matching (PMM) for the procedure. We created five imputed datasets and calculated the pool results in the regression models. We used the ‘mice’ package of R^33^ for these calculations. We rely primarily on the average marginal effects (AME). In a logistic regression model, the AME measures the average change in the predicted probability of the outcome variable (y) occurring for a one-unit increase in a predictor variable $x_i$, holding all other variables constant. Logistic regression probabilities are non-linear, so the impact of $x_i$ depends on the values of all predictors. AME averages these effects across the data for an overall summary. For example, if the AME for $x_i$ is 0.05, it means that, on average, a one-unit increase in $x_i$ leads to a 5-percentage-point increase in the probability of y. AME serve as a more comparable quantity of interest across different regression models^34^.

## Results

One thousand participants took part in the 2022 vignette experiment survey. Of these, 769 respondents were regular Facebook users, had a Google account, and still were members of the online panel. These people could share their social media data in the 2023 data donation survey. Of this group, 62% consented to share their data, and 21% eventually donated their data. 21% is a relatively high number compared to previous research^20,22^, but it is worth bearing in mind that these respondents were among the more active online panel members. Our sample can be divided by who dropped out in which part of the procedure. 19% of respondents were invited to the study but did not click on the platform, and a further 7% clicked on the platform but did not view the consent form or the detailed technical description of the survey. Of the respondents invited to the sample, 13% stopped the survey at the consent form section. We considered the people who dropped out in these stages as those who were unwilling to participate in the data donation study. All of these people got the invitation letter, so even those who did not click on the data collection platform had information about the design and objective of the study (the invitation letter is available in the supplementary). 17% accepted the consent form but did not start the data upload. Perhaps the most challenging part of the research was the data upload, which required first downloading the social media data from at least Facebook and Google Takeout in the requested format and then uploading the downloaded data to the research company’s dedicated server. At this point, 24% of our sample dropped out.

The average willingness indicator in the vignette experiment study was 5.1. However this value was significantly different between those who stopped the procedure at different levels and those who eventually completed the data donation (Anova *p* <0.01).

As shown in Table 2, up to the point where no consent is given, we tend to see respondents with lower willingness averages among those who gave consent but did not ultimately start the uploading process and even higher among those who started uploading but did not complete it. The highest willingness values were measured among those who completed the whole data transfer. Those who started but did not finish the upload were, therefore, not only not completing it because they had some technical problem with the upload but also had a fundamentally lower willingness to transfer data. The willingness value explains 10.6% of the completion status. We calculated the mean willingness value for the different procedure stages with all vignettes instead of the proximity-based version we used in the main analysis (Table S1). The tendencies were the same, but the $\eta ^{2}$ value was smaller (0.08), which means that the proximity-based measure of willingness predicts better the actual behavior.

**Table 2:** Average willingness score of the respondents who stopped the procedure at different levels and those who eventually completed the data donation (Anova *p* < 0.01, $\eta ^{2}$ = 0.11).

Category	Mean	N	CI
Invited, but did not click on the platform	4.41	146	3.83–4.98
Clicked on the platform	3.46	54	2.52–4.40
Not consented	3.56	100	2.87–4.25
Consented, but did not start the uploading process	4.93	130	4.32–5.54
Started the uploading process, but did not finish	5.39	190	4.89–5.89
Finished the process	7.19	166	6.65–7.72

**Fig. 1:**
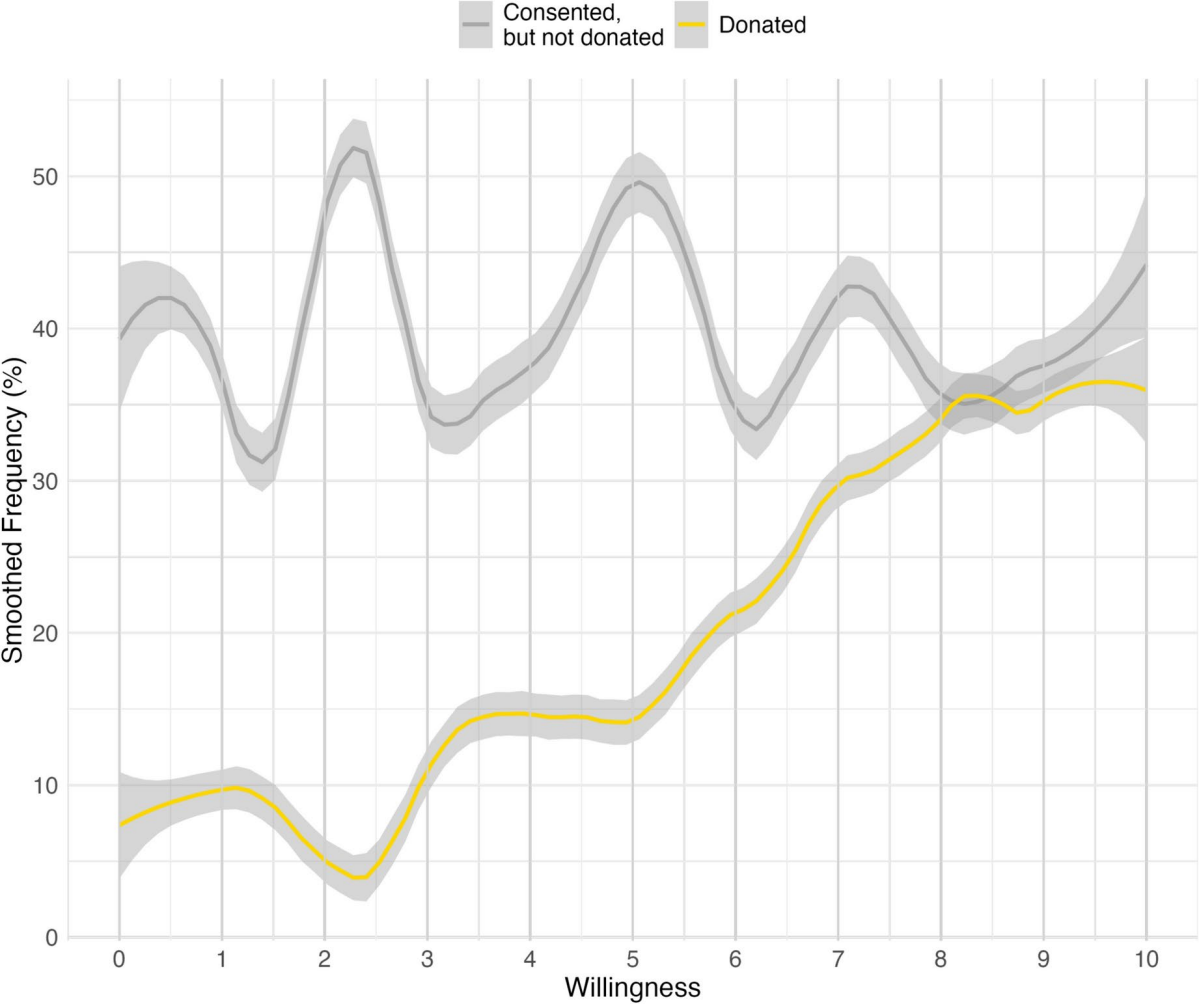
Participation for different willingness scores.

The correlation ($\eta$) between the willingness score and whether people donated their data is 0.28. The same $\eta$ value between those who at least consented and the willingness is 0.25. The willingness correlates slightly more with the donation than with the consent. This result reflects well on the mean differences in Table 2. To better understand the relationship between consent, donation, and willingness, we analyzed how the willingness score of those people looked like, who consented to the second study but did not donate their data at the end. As the willingness score was a continuous measure but with a limited number of categories, we calculated smoothed values with a 1-point span to better present the relationship between willingness and the outcome of the donation study. Figure 1 shows the smoothed frequency of those who donated and those who consented but did not donate contrasted to the willingness score. Of those who gave zero to the willingness question, 7.7 percent finally donated their data, so it was possible to do the data donation study even within these people. However, among those who gave the highest willingness score (10), 34.7 percent donated their data. The real increase in the donation ratio starts from around willingness score 5, and we see a significant increase from 6 to 7 and 7 to 8. The donation level is the same for those above eight willingness scores. The ratio of those who consented but did not donate has no clear trends regarding the willingness score. So, from a predictive validity perspective, it seems that actual donation works better than just consent on donation.

**Fig. 2:**
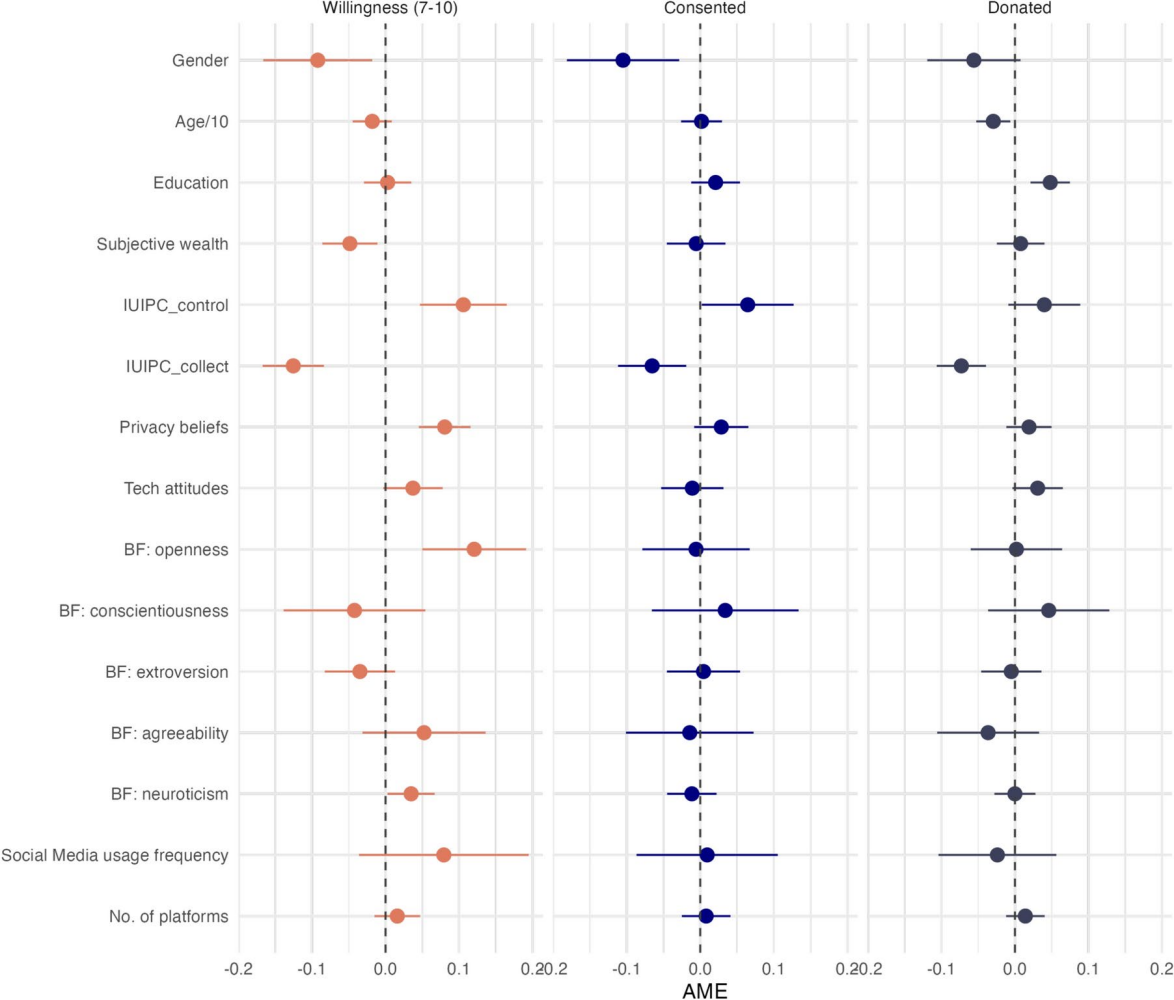
Results from the models predicting willingness and actual behavior (pooled results).

Our second research question focused on the equivalence of the drivers of willingness and actual behavior. Based on the analysis above, we coded the value of 7–10 willingness to participate as one and the lower value as zero. We also tested different cut-off values (6 and 8) and models without categorization for robustness checks (see later). In the second model, the dependent variable was whether or not the subject of the pre-study consented to participate in the data donation study. In the third model, we used the actual donation as the dependent variable. The explanatory power of the willingness and donation models was very similar, with a pseudo-$R^{2}$ statistic of around 0.13 and 0.1. The pseudo-$R^{2}$ was lower for the consent model, only 0.04. The independent variables also had different impacts on the three outcomes. The results of the regression models are shown in Fig. 2 and Table S2. We also calculated if the AME values of the same variables differ in the models (Table S3). Gender was significant at the 0.05 level for willingness to participate (women were more likely to participate) and for consent in the data donation study and at 0.1 level for actual donation. The AME values of gender did not differ significantly between the models (Table S3). There was no significant impact of age on willingness and consent, but we found a small (AME: – 0.03) effect on donation. This means that the donation rate decreased by an average of 3% with every 10 years of age. However, the difference between the models’ AME values was not significant. Education was not significant in willingness to share or consent to participate, but it was crucial in actual data donation. Based on the marginal prediction, the donation probability was less than 10% for those with the lowest education level and more than 30% for those with university degrees. The difference in the AME value of education between the willingness and the donation model was significant (p=0.03). Subjective income worked oppositely. This variable was not a significant predictor of consent and donation but was negatively associated with willingness. The AME value of willingness was -0.05, so the model predicted an average of 5% probability decrease per subjective income category (this variable had five categories). The AME values were not significantly different for willingness and consent but were significant between willingness and donation (p = 0.02). The data-control dimension of the privacy control index (IUIPC) had an AME value between 0.06 and 0.11 in the three models. These values did not differ significantly, although the effect was significant for the willingness but not for the consent and donation. The data collection dimension of the privacy control index was significant in all the models, affecting both willingness and consent and actual data sharing. Thus, those who fear that tech companies would collect too much personal data about them theoretically and also practically are more likely to drop out of such a study. However, the effect size was not the same; the absolute value of the AME value was higher for willingness (– 0.13) than for consent and actual donation (-0.07). Figure S5 presents the predicted marginal probability for the three models. Attitudes towards adequately storing and protecting personal data were still essential predictors at the willingness level but not on the consent and donation levels. The 0.08 AME value of willingness was significantly higher than the 0.03 value of consent and the 0.02 value of donation. The main results of the privacy question were that they had a stronger effect size in the case of willingness and a lower effect size for actual consent and donation. None of the questions in the Big Five inventory were significantly related to whether people consented to share their data or to donate it. However, openness was a crucial factor in the willingness to share. As presented in Figure S5, the predicted willingness with a 7–10 cut-off was less than 20% for those with low openness but more than 65% for those with high openness. However, for consent and donation, we did not see any effect. The frequency of use of social media platforms was not significant in any of the models, nor was the number of platforms used at the 5% significance level. We tested the robustness of our results with many alternative models. We changed the cut-off value of willingness to 6–10 and 8–10, and we also tested the 7–10 cut-off value with a willingness based on all vignettes, not just the proximity ones. We also tested willingness without any cut-off on its continuous form, and we tested Linear Probability Models instead of the logistic regression. The results (Figs. S6–S7) were robust for the alternative model specifications.

## Discussion

Prior research suggested that intentions expressed by participants in vignette experiments may poorly correspond to their actions in real-life situations^2,3^. In this study, we used a vignette experiment to plot key predictors of willingness to share data. We returned to the same respondents nine months later and asked them to donate their social media data, similar to the experimental situation. In our study 62% consented to share their data, and 21% donated it At the individual level, the correlation was found to be medium ($\eta$: 0.28) with the actual donation, which is consistent with prior results^16^. The moderate correlation can be due to response biases, the time spent between the two actions^35^ or discrepancies between the two scenarios. It was also an open question whether we found a stronger correlation between the willingness and consent to participate in the data donation study or the actual donation. We found evidence for the latter. Consenting but not donating at the end did not show a clear correlation with the willingness score. We can assume that some of the respondents were just curious about the study, and when they realized how complex the process was, they did not proceed. Higher initial willingness has some “protection” power against this attrition type. We also expected that those almost sure to share their data will not all share it in practice. Unexpectedly, however, even among those who said they were almost sure they would not share their social media data, data sharing eventually occurred. A moderate correlation of willingness and behaviour at the individual level is not necessarily a significant problem because these experimental studies mainly focus on the determinants of data sharing^1^. Our results are not reassuring in this respect either. Willingness was explained primarily by softer, attitudinal variables. When only demographics and platform usage variables were included in the model, the $R^{2}$ value of 0.14 dropped to around 0.03. In contrast, the model that included only attitude variables achieved $R^{2}$ values above 0.13. Two of the Big-5 questions were significant in modeling willingness, and, in particular, openness had a strong effect with an AME value of 0.12. In contrast, the Big-5 traits did not play any role in giving consent and actual data donation. Although the Big-5 inventory is one of the most prevalent and extensively utilized frameworks in personality studies^36^, our results indicate the potential presence of biases in these measures, possibly linked to social desirability. Instead of soft variables, hard demographic variables played a stronger role in real data sharing. A model with only demographic variables achieved an $R^{2}$ value of nearly 0.05 in the real data-sharing model, compared to a value of around 0.03 in the case of willingness. Both age and educational attainment were crucial variables in the actual data donation, although these variables were not significant when willingness to participate was measured. The difference in the AME value of education between the willingness and the donation models was significant (p = 0.03), but for age, it was not significantly different. The actual data donation was a complex process. Participants had to download the relevant data types from their social media platforms in the appropriate format and then upload the downloaded files to a server. The process was supported with a video explaining the data-downloading process and a textual description. However, social media sites regularly change the structure of their pages and even use several different page designs simultaneously, based on our experience. Thus, someone may have had to use a different way of downloading data than the one described. In addition, following the exact download protocol may have been challenging for people who are more insecure about using the internet. Participants may have asked for help by phone if they got stuck in the process, but there may also have been some who were intimidated by the complexity of the task and gave up. We expected internet usage skill (ATI) to be significantly related to actual data sharing, however, this variable did not prove significant in the models. The problem may be that ATI focuses not on proficiency but on interest in new technologies. Education and age, on the other hand, may have been a latent measure of technology proficiency, which causes higher data donation rates for younger and higher-educated individuals. It may be worthwhile to measure Internet usage knowledge with more specific questions in similar surveys.

Some of our results are also relevant to vignette experiments that examine willingness to participate in data sharing. We found evidence for the internal validity of the vignette experiment. In the original study, we found that incentives were the most critical factor in willingness to donate. When we calculated the willingness score for this study and used the willingness score with the highest incentive, we got a stronger correlation with the actual donation compared to the willingness calculation when we used all available vignettes. Another problem in these vignette experiments is that if researchers want to dichotomize the outcome variable, what boundaries should they set for doing so? Our results show that the actual donation level was the same for willingness scores between 8 and 10. However, from a modeling perspective, the results were robust whether we chose six, seven, or eight as a cut-off value.

To conclude, in line with some of the earlier findings^13–15^, willingness measures in conjoint and vignette experiments may only moderately reflect actual real-life behavior. Additionally, and potentially more troubling, the factors influencing each may not coincide. Willingness to share was better explained by soft attitudinal variables, while in contrast, actual data sharing was associated with harder demographic variables. It could be argued that despite the discrepancies between willingness, actual sharing and their determinants, the results of the experimental manipulations may still be valid. We recommend that future studies design experiments capable of reliably comparing the validity of treatment effects between stated willingness and actual behavior.

One explanation for our findings can be that participation in the data donation study may pose a high personal risk to the individuals. Based on the theory of planned behavior^4^ this likely made the intention-behavior relation weaker. Our findings suggest that this high perceived risk can also moderate the relationship between predictors of willingness and actual behavior. Time can also play a role. In the study of Keusch et al.^16^, participants were asked to donate immediately following the survey, whereas, in our study, there was a nine-month gap before the donation request. This difference in timing may account for the greater alignment between willingness and actual donation behavior found in the Keutsch et al. study compared to ours.

Our study is not without limitations. First, discrepancies between survey responses and behaviour may partially originate from the differences between scenarios described in the vignettes and the requests used in the actual project. It is also possible that the time between the two actions was too long. Research on the role of time intervals in test-retest reliability in health studies indicates that longer time intervals - exceeding a few months - significantly increase the risk of changes in participants’ status or attitudes^35,37^. This may affect other variables used in the study. However, we do not expect strong changes in people’s wealth, personality traits, affinity for technology or privacy beliefs within a few months. Platform usage could have changed for some participants, however considering that the vast majority of study participants were relatively heavy users, we do not perceive this as a significant concern. We advise future studies to conduct similar validation with shorter time intervals. Second, the two requests were not fully identical. The main difference between them was that the actual request contained more information than the descriptions in the vignette study. In the vignette study, we prioritized shortness and simplicity. In contrast, the actual request required a detailed and transparent explanation to provide participants with sufficient information for making an informed decision. It is possible that discrepancies between willingness and behaviour were partially due to invitees receiving more detailed information in the actual request. Third, it is unclear how broadly our results apply to other countries. Also, the online panel utilized in this study was not selected through probability methods. Nevertheless, despite that our sample is not perfectly representative of the online population, the composition of these panels is expected to closely mirror the types of participant groups that researchers are likely to select in data donation studies. Future studies are encouraged to replicate our validation efforts in other cultures and settings. Importantly, the type of behavior and the context of the experiment seem to matter a lot and explain inconsistencies in the literature, thus our findings cannot necessarily be generalized to meaningfully different willingness measures.

This study contrasted willingness to share social media data in a vignette experiment with actual sharing behavior using within-person comparisons. Our findings suggest that in high-risk scenarios such as a data donation project, estimates may be biased both for descriptive and correlational analysis. Future research should mitigate the discrepancies between self-reported behaviors in survey experiments and actual real-world actions by crafting realistic hypothetical situations, implementing strategies to minimize participants’ perceived risks and concerns associated with the tasks, and accounting for social desirability responding.

## Supplementary Information

Supplementary Information.

## Data Availability

The data used in the analysis is available here: https://osf.io/kyhp5/files/osfstorage
