# Supplementary material for: Validating a willingness to share measure of a vignette experiment using real-world behavioral data
Source: Sci Rep. 2025 Mar 18;15:9319. doi: 10.1038/s41598-025-92349-2 (PMC11920269; doi:10.1038/s41598-025-92349-2)
Supplement: Supplementary file 1 — Supplementary Information. [file 41598_2025_92349_MOESM1_ESM.pdf]

Validating a Willingness to Share Measure of a Vignette Experiment Using Real-World Behavioral Data

Supplementary information

Contents

1 The manipulated text (The bold parts were the manipulated part of the experiment) 2

2 Invitation to the data donation study 2

3 Detailed operationalization of independent variables 6

4 Supplementary tables and figures 7

1 The manipulated text (The bold parts were the manipulated part of the experiment)

"The various social media sites and platforms (Facebook, Instagram, Twitter, Google) allow users to view and even download information and data about themselves stored on the site. This data is very valuable from a scientific point of view since it captures behavioral patterns not observed elsewhere. Imagine a situation in which you are asked by the Social Science Research Centre to participate in a survey. You are invited to fill in a questionnaire, and you are asked to share your **Facebook** data, **excluding your private messages and pictures/videos**. Downloading the data to your computer and uploading it to the research page would take **less than 1 hour**. For participating in the research, you would receive **3000 HUF** and **a personalized report on your social media usage compared to the rest of the Hungarian internet population**. Once uploaded, the data would be anonymized and only analyzed for research purposes.

Please indicate on a scale of 0-10 how likely would you be to participate in such research! 0 indicates not likely at all, and 10 indicates very likely."

2 Invitation to the data donation study

Társadalomtudományi Kutatóközpont
Magyar Tudományos Akadémia Kiváló Kutatóhely

ELKH Eötvös Loránd Kutatási Hálózat

netpanel

Dear <NAME>!

Would you like to participate in scientific research?
We are looking for volunteer participants for **social science research** on internet and social media use.

The research is organised by the **Centre for Social Sciences** with the support of Netpanel.

What happens in the research?

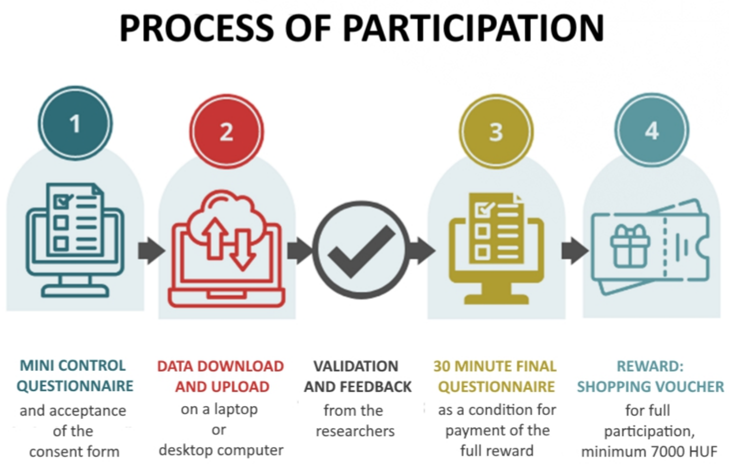

Details:

1. Mini control questionnaire: applicants are first asked to fill in a **very short control questionnaire and to agree to a consent form**.

2. Data upload task: this is followed by an **independent task that can be completed on a laptop or desktop computer** (approximately 1-2 hours).

3. Final questionnaire: as a final step, after a few days of monitoring, participants are asked to complete an **online questionnaire of about 30 minutes**.

Until when can you participate? We expect our research to end by May 2023.

Fig. S1: First page of the invitation to the data donation study

Dear <NAME>!

Would you like to participate in scientific research?
We are looking for volunteer participants for **social science research** on internet and social media use.

The research is organised by the **Centre for Social Sciences** with the support of Netpanel.

What happens in the research?

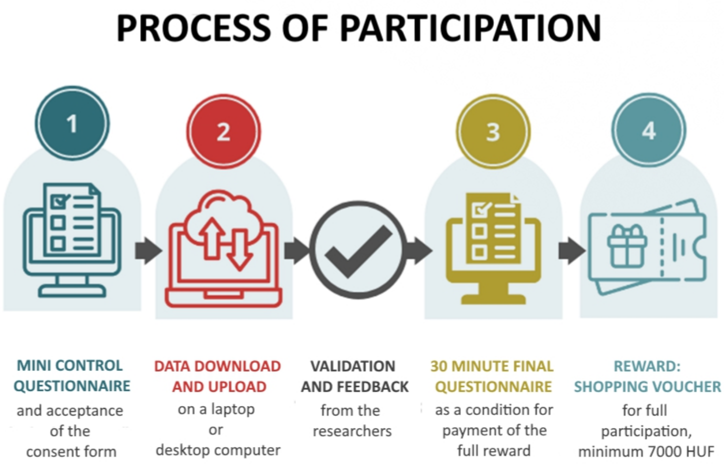

Details:
1. Mini control questionnaire: applicants are first asked to fill in a **very short control questionnaire and to agree to a consent form**.

2. Data upload task: this is followed by an **independent task that can be completed on a laptop or desktop computer** (approximately 1-2 hours).

3. Final questionnaire: as a final step, after a few days of monitoring, participants are asked to complete an **online questionnaire of about 30 minutes**.

Until when can you participate? We expect our research to end by May 2023.

Fig. S2: Second page of the invitation to the data donation study

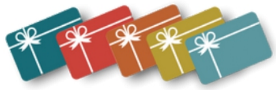

Why participate? Guaranteed voucher worth HUF 7.000!

As well as helping to make scientific research a success, volunteers are guaranteed a reward **if they take part in all three research phases**.

The reward will be worth **at least HUF 7000 net and will be paid in the form of a voucher** to participants who complete the required tasks in full.

By completing extra tasks, you can earn additional rewards (up to a maximum of HUF 3000 extra in total)!

The vouchers can be spent at a popular national hypermarket chain with a large network of stores. Payments are ongoing for those who complete all three research phases.

IMPORTANT INFORMATION

>>> Participation is **voluntary**,
>>> fully online, no need to be present in person.

>>> Your participation in the research will be rewarded with a **fixed bonus** (if the conditions are met).

IMPORTANT: In phase 2 of the survey, personal data is transferred: this data relates to the use of the internet browser and the use of certain social media platforms (e.g. Facebook, Instagram, Tiktok).

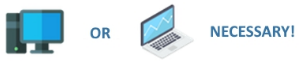

You can only participate in phase 2 of the survey from **a laptop or desktop computer**. Please only volunteer if you will be able to do so technically.

Take part, collect your reward and spend it where you want!

I PARTICIPATE

Start

Thank you,

the MTA researchers and the Netpanel team

Fig. S3: Third page of the invitation to the data donation study

3 Detailed operationalization of independent variables

Gender is a binary variable that assigns 0 to males and 1 to females. We measured the highest level of education of respondents with six categories: from "primary" to "university diploma. We operationalized age with the year of birth. Subjective wealth was measured with five categories, where the highest means that they live without financial problems, and the lowest means that they live in deprivation.

To control social media usage, we used two variables. The first variable was the frequency of social media usage on a 1 to 5 scale, where the lowest means never, and the highest means daily. Here we calculated the maximum of Facebook, Twitter, Instagram, Youtube and TikTok usage. The second variable was the number of social media platforms, on which the respondent is active. Here we asked about the following platforms: Facebook, Instagram, Twitter, Youtube, Linkedin, TikTok, and Spotify.

To measure respondents' privacy concerns, we used the Internet Users' Information Privacy Concerns (IUIPC) scale [1]. We applied a confirmatory factor model to extract the three latent dimensions behind the eight validated items. According to our analyses, the model with the three latent variables fit the data well (CFA:0.99, RMSEA: 0.068). Out of these three dimensions, in the analysis, we only used the 'control' and 'collection' dimensions of the scale and omitted the 'awareness' one, as it highly correlates with the 'control' in both samples. High values of these dimensions mean high control over personal information and concerns about the collection of personal data by companies.

For measuring privacy concerns, we calculated the principal component of the following two variables (measured on a 1 to 7 scale):

- "Most businesses handle the personal information they collect about consumers in a proper and confidential way."

- "Existing laws and organizational practices provide a reasonable level of protection for consumer privacy today."

High values here mean high trust in how businesses and organizations protect consumer data.

To measure the respondents' affinity for technology, we used the 9-item version of the Affinity for Technology Interaction Scale (ATI [2]). We calculated the mean of the items after reverse coding the needed items. The value of Cronbach's Alpha was 0.84 in the study. High values here mean a high affinity for technology.

The last group of independent variables was the Big Five (BFI-15) inventory [3]: Extraversion, Agreeableness, Conscientiousness, Neuroticism, and Openness. We used the 15-item version of the scale and applied a confirmatory factor model to extract the dimensions. The tested factor model fit the data (CFA: 0.99, RMSEA: 0.034). We had to drop out the reversed coded items from the models because of their poor fit.

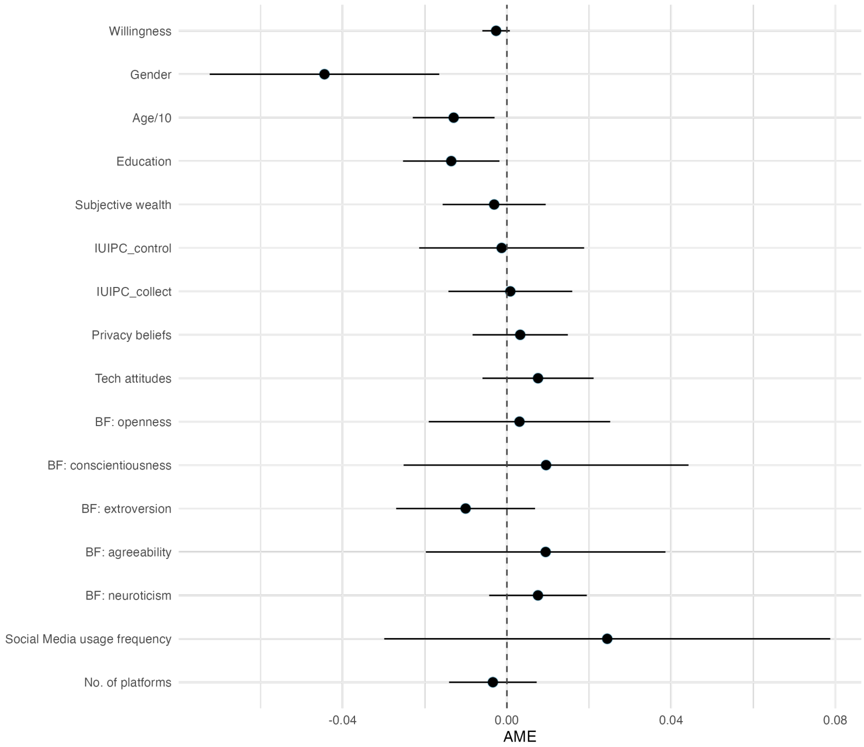

Fig. S4: Results of the model predicting attrition from the online panel - Logistic model

4 Supplementary tables and figures

Table S1. Average willingness score (calculated with all vignettes) of the respondents who stopped the procedure at different levels and those who eventually completed the data donation (Anova $p < 0.01$, $\eta^2 = 0.08$.)

Category	Mean	N	CI
Invited, but did not click on the platform	3.92	146	3.38 - 4.46
Clicked on the platform	2.98	54	2.10 - 3.86
Not consented	3.16	100	2.51 - 3.81
Consented, but did not start the uploading process	4.25	130	3.68- 4.82
Started the uploading process, but did not finish	4.57	190	4.10 - 5.04
Finished the process	6.16	166	5.65 - 6.66

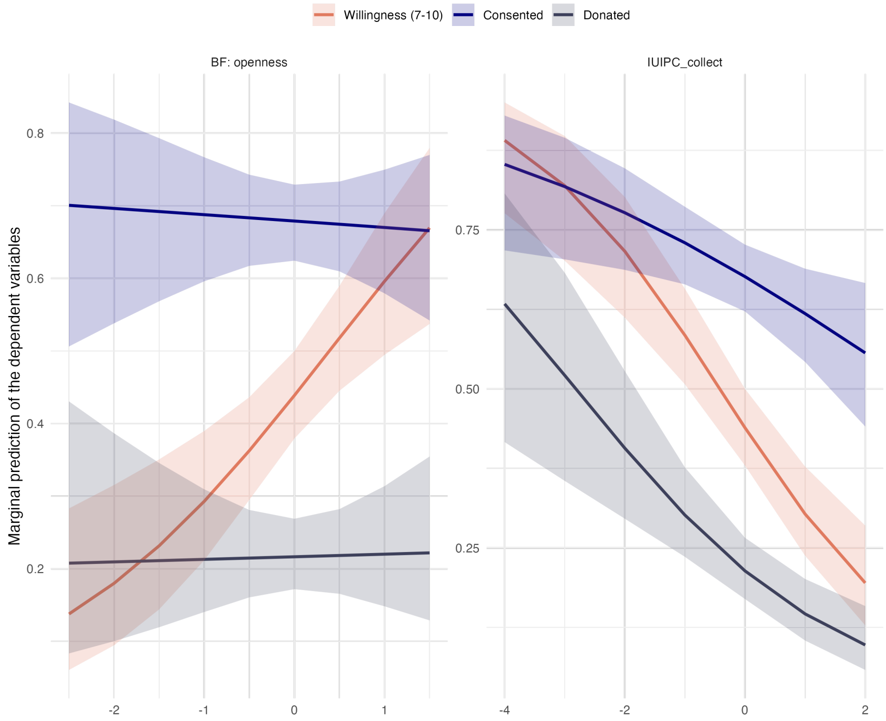

Fig. S5: Marginal predictions of willingness, consent and donation - Logistic models (1 imputation selected)

Table S2. Results from the models predicting willingness, consent, and donation behaviors

	Willingness (7-10)				Consented				Donated			
Predictors	AME	OR	CI	p	AME	OR	CI	p	AME	OR	CI	p
(Intercept)		0.16	0.01-3.78	0.25		1.29	0.11-16.03	0.84		0.12	0-2.86	0.18
Gender	-0.09	0.64	0.45-0.92	0.02	-0.11	0.63	0.45-0.89	0.01	-0.06	0.69	0.46-1.05	0.08
Age	-0.02	0.92	0.81-1.05	0.19	0.00	1.01	0.89-1.14	0.90	-0.03	0.82	0.71-0.96	0.01
Education	0.00	1.01	0.87-1.18	0.86	0.02	1.10	0.95-1.27	0.21	0.05	1.37	1.15-1.65	0.00
Subjective wealth	-0.05	0.79	0.66-0.95	0.01	-0.01	0.98	0.82-1.17	0.79	0.01	1.05	0.85-1.31	0.64
IUIPC_control	0.11	1.66	1.23-2.27	0.00	0.06	1.33	0.96-1.85	0.09	0.04	1.30	0.94-1.83	0.12
IUIPC_collect	-0.13	0.55	0.44-0.68	0.00	-0.07	0.75	0.61-0.93	0.01	-0.07	0.62	0.49-0.79	0.00
Privacy beliefs	0.08	1.47	1.22-1.79	0.00	0.03	1.13	0.96-1.35	0.14	0.02	1.14	0.91-1.42	0.26
Tech attitudes	0.04	1.20	0.98-1.46	0.08	-0.01	0.95	0.79-1.15	0.62	0.03	1.23	0.98-1.55	0.08
BF: openness	0.12	1.79	1.26-2.56	0.00	-0.01	0.98	0.70-1.37	0.88	0.00	1.01	0.67-1.54	0.95
BF: conscientiousness	-0.04	0.82	0.51-1.32	0.40	0.03	1.16	0.74-1.84	0.52	0.05	1.36	0.77-2.40	0.29
BF: extroversion	-0.03	0.85	0.67-1.07	0.16	0.00	1.02	0.81-1.29	0.87	0.00	0.97	0.74-1.27	0.81
BF: agreeability	0.05	1.29	0.85-1.96	0.23	-0.01	0.94	0.64-1.38	0.74	-0.04	0.78	0.49-1.27	0.31
BF: neuroticism	0.03	1.18	1.00-1.40	0.05	-0.01	0.95	0.82-1.11	0.51	0.00	1.00	0.83-1.20	1.00
Social Media usage frequency	0.08	1.47	0.84-2.58	0.18	0.01	1.04	0.69-1.59	0.85	-0.02	0.85	0.50-1.46	0.56
No of platforms	0.02	1.08	0.93-1.26	0.32	0.01	1.04	0.89-1.20	0.64	0.01	1.10	0.92-1.31	0.30
N	769				769				769			
R^2 Tjur	0.133				0.04				0.096			

Table S3. Comparing AME Values (Z Test) - P Values

Predictors	Willingness vs Consented	Willingness vs Donated
Gender	0.81	0.46
Age	0.30	0.52
Education	0.44	0.03
Subjective wealth	0.12	0.02
IUIPC_control	0.34	0.09
IUIPC_collect	0.05	0.05
Privacy beliefs	0.04	0.01
Tech attitudes	0.10	0.81
BF: openness	0.01	0.01
BF: conscientiousness	0.27	0.16
BF: extroversion	0.26	0.34
BF: agreeability	0.27	0.10
BF: neuroticism	0.05	0.10
Social Media usage frequency	0.35	0.14
No of platforms	0.73	0.93

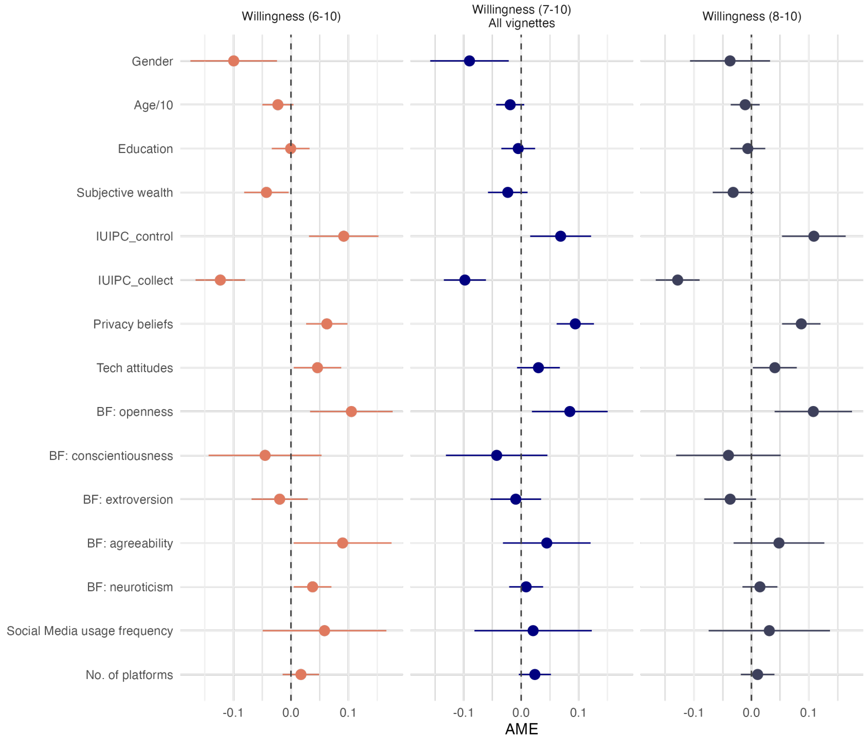

Fig. S6: Results from the alternative models predicting willingness - Logistic models (pooled results)

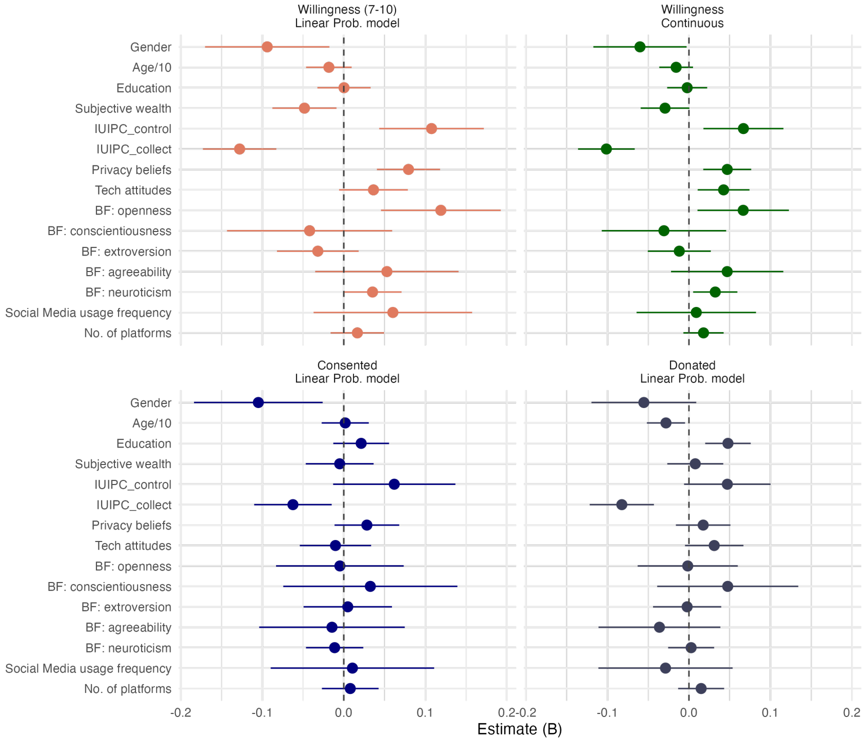

Fig. S7: Results from the alternative models predicting willingness, consent and donation - Linear models (pooled results)

References

[1] Malhotra, N.K., Kim, S.S., Agarwal, J.: Internet Users' Information Privacy Concerns (IUIPC): The Construct, the Scale, and a Causal Model. Information Systems Research **15**(4), 336–355 (2004) https://doi.org/10.1287/isre.1040.0032 . Publisher: INFORMS. Accessed 2023-02-19

[2] Franke, T., Attig, C., Wessel, D.: A Personal Resource for Technology Interaction: Development and Validation of the Affinity for Technology Interaction (ATI) Scale. International Journal of Human-Computer Interaction **35**(6), 456–467 (2019) https://doi.org/10.1080/10447318.2018.1456150

[3] Lang, F.R., John, D., Lüdtke, O., Schupp, J., Wagner, G.G.: Short assessment of the Big Five: robust across survey methods except telephone interviewing. Behavior Research Methods **43**(2), 548–567 (2011) https://doi.org/10.3758/s13428-011-0066-z . Accessed 2024-02-15
